# Supplementary material for: Codoped porous carbon nanofibres as a potassium metal host for nonaqueous K-ion batteries
Source: Nat Commun. 2022 Aug 20;13:4911. doi: 10.1038/s41467-022-32660-y (PMC9392754; doi:10.1038/s41467-022-32660-y)
Supplement: Supplementary file 1 — Supplementary Information [file 41467_2022_32660_MOESM1_ESM.pdf]

Supplementary Information

Codoped porous carbon nanofibres as potassium metal host for nonaqueous K-ion batteries

Siwu Li,[1] Haolin Zhu,[1] Yuan Liu,[2] Zhilong Han,[1] Linfeng Peng,[1] Shuping Li,[1] Chuang Yu,[1] Shijie Cheng[1] and Jia Xie[1*]

[1] State Key Laboratory of Advanced Electromagnetic Engineering and Technology, School of Electrical and Electronic Engineering, Huazhong University of Science and Technology, Wuhan, 430074, P. R. China.

[2] Key Laboratory for Renewable Energy, Beijing Key Laboratory for New Energy Materials and Devices, Beijing National Laboratory for Condensed Matter Physics, Institute of Physics, Chinese Academy of Sciences, Beijing 100190, China

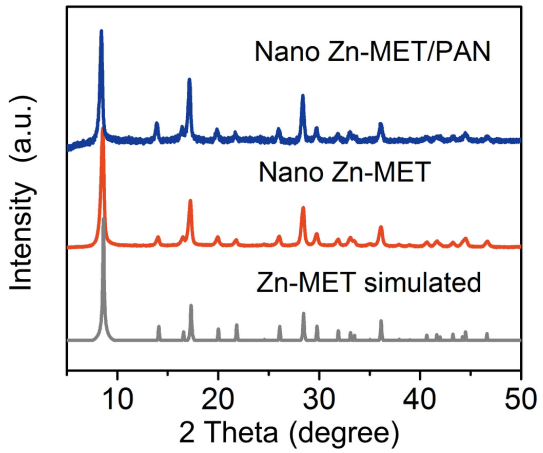

Supplementary Figure 1. Phase characterization of MOF-based materials. XRD patterns of nano MET-6 (Zn-MET) particles and their PAN composite.

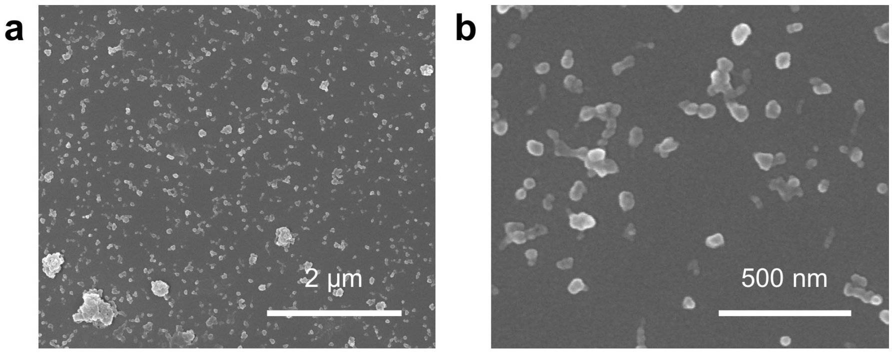

Supplementary Figure 2. Morphology of the synthesized MOF particles. SEM images of (a) nano-sized MET-6. (b) The enlarged image of (a).

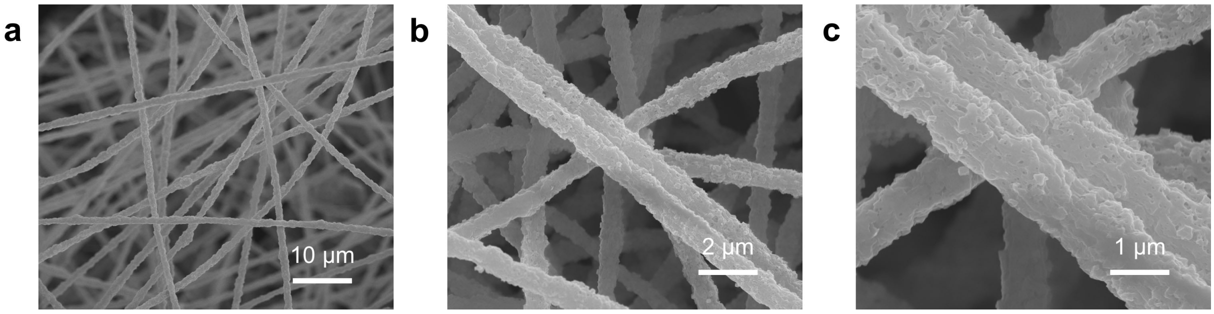

Supplementary Figure 3. Morphology of the synthesized MOF/PAN fibers. SEM images of nano-MET-6/PAN fibers under different magnifications.

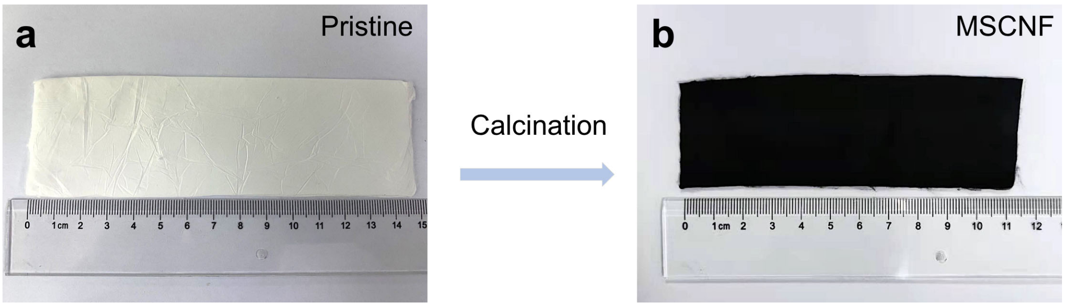

Supplementary Figure 4. Digital photos of nano-MET-6/PAN and MSCNF. (a) pristine nano-MET-6/PAN film and (b) its corresponding product MSCNF after calcination at 600 °C.

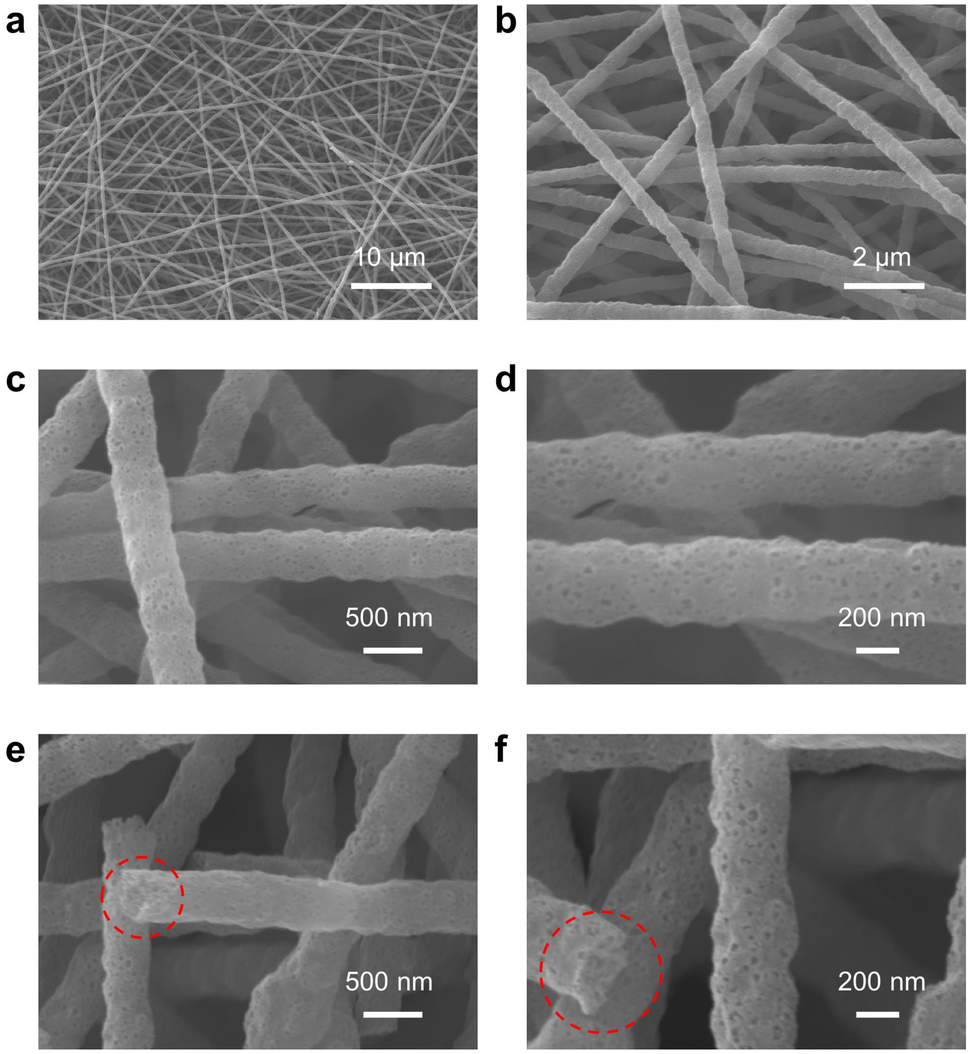

Supplementary Figure 5. Morphology of MSCNFs. SEM images of MSCNFs under different magnifications. The red dotted circles in (e) and (f) highlight the porous structure inside the MSCNFs from the cross-section of a single fiber.

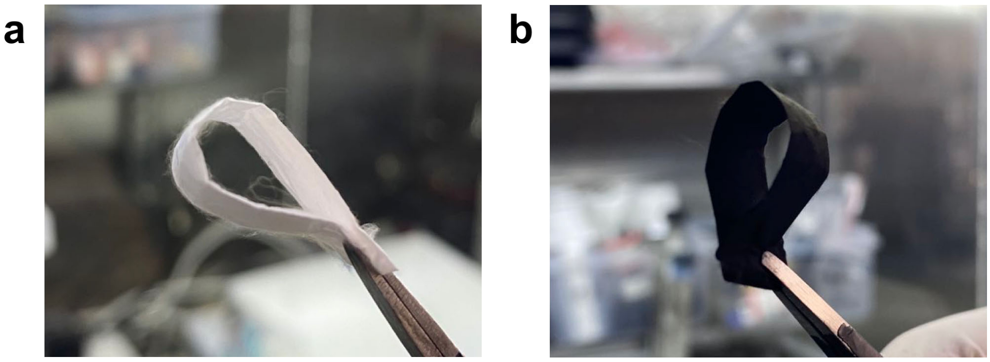

Supplementary Figure 6. Flexibility demonstration. Digital photos of (a) pristine nano-MET-6/PAN film and (b) MSCNFs in a bent state.

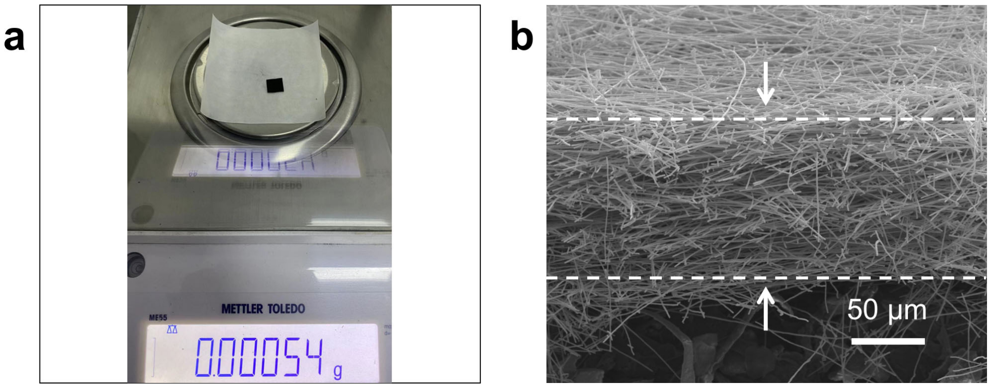

Supplementary Figure 7. Weight and morphology of MSCNFs. (a) Weight of MSCNFs with an area of 1 cm^2. (b) SEM image of the cross-section view of MSCNFs.

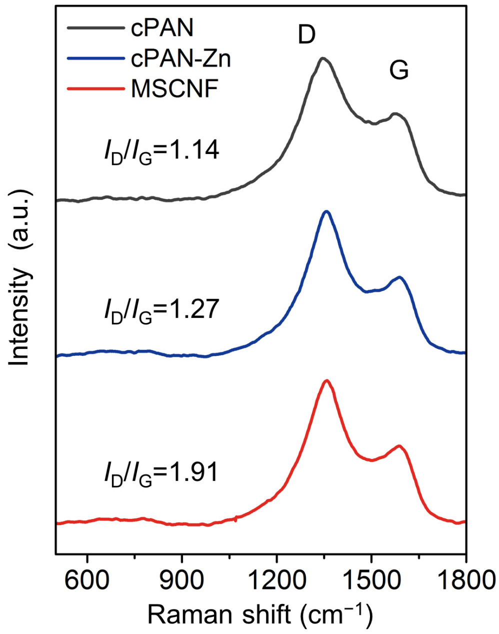

Supplementary Figure 8. Defect evaluation of different carbon-based hosts. Raman spectra of cPAN, cPAN-Zn and MSCNF.

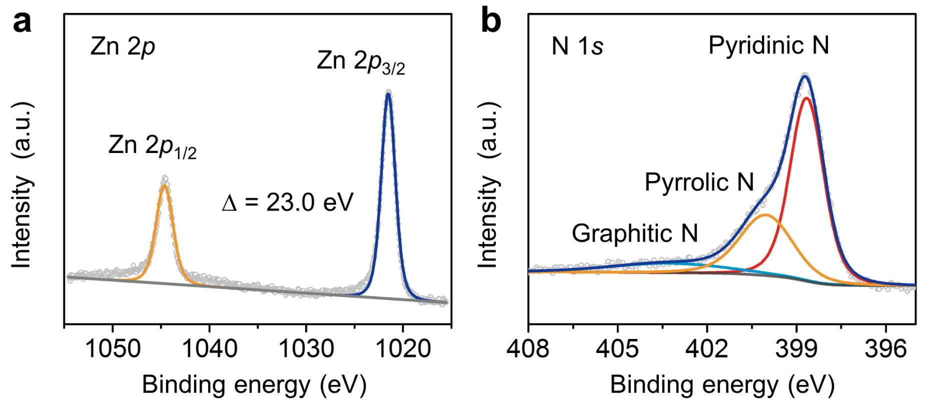

Supplementary Figure 9. Chemical analysis of MSCNFs. XPS spectra of (a) Zn 2*p* and (b) N 1*s* from MSCNFs.

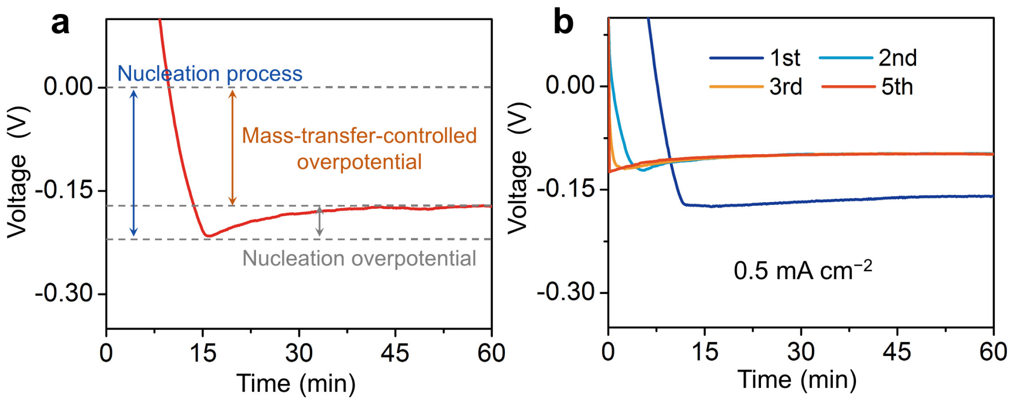

Supplementary Figure 10. Potassiophilicity evaluation. (a) K nucleation behavior on a host. (b) Voltage profiles of K plating on MSCNFs at different cycles at a current density of 0.5 mA cm^{-2}.

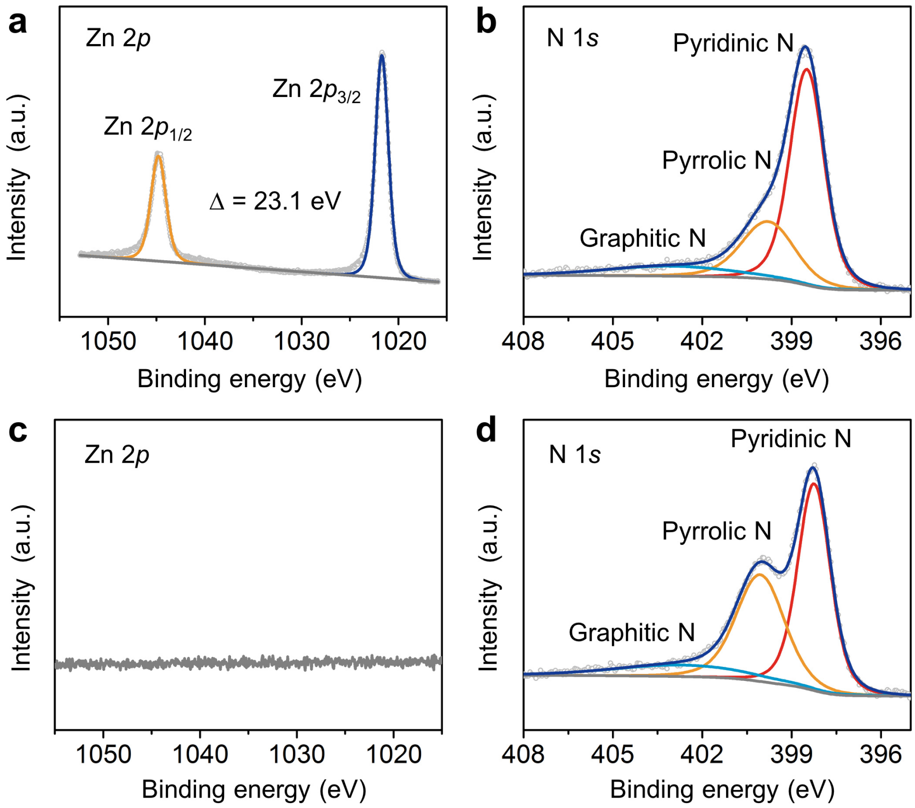

Supplementary Figure 11. Chemical analysis of different nanofibers. XPS spectra of (a) Zn 2*p* and (b) N 1*s* from cPAN-Zn. XPS spectra of (c) Zn 2*p* and (d) N 1*s* from cPAN.

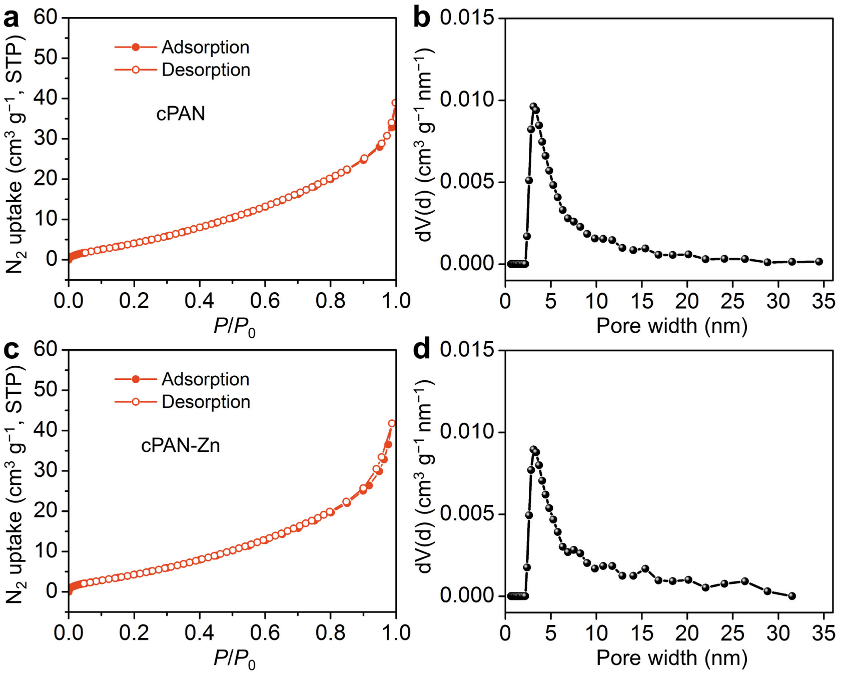

Supplementary Figure 12. Pore size evaluation of different materials. N_2-77 K adsorption isotherms of (a) cPAN and (c) cPAN-Zn, and (c, d) corresponding pore distribution.

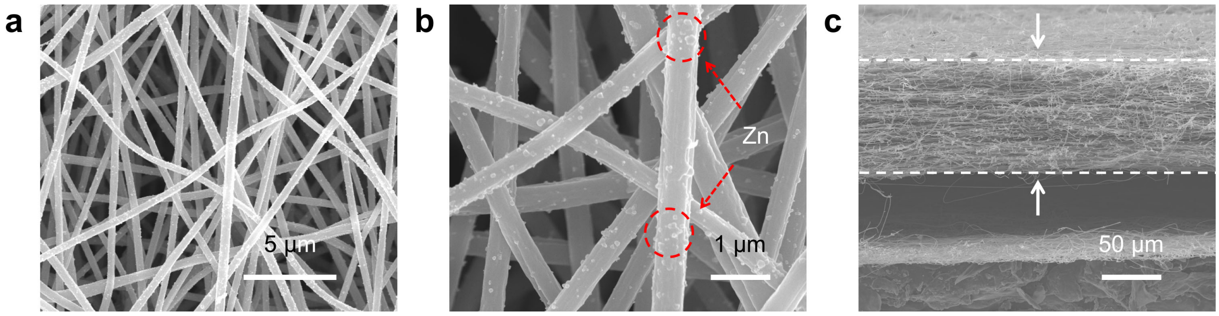

Supplementary Figure 13. Morphology of cPAN-Zn. SEM images of (a, b) front view and (c) cross-section view of cPAN-Zn.

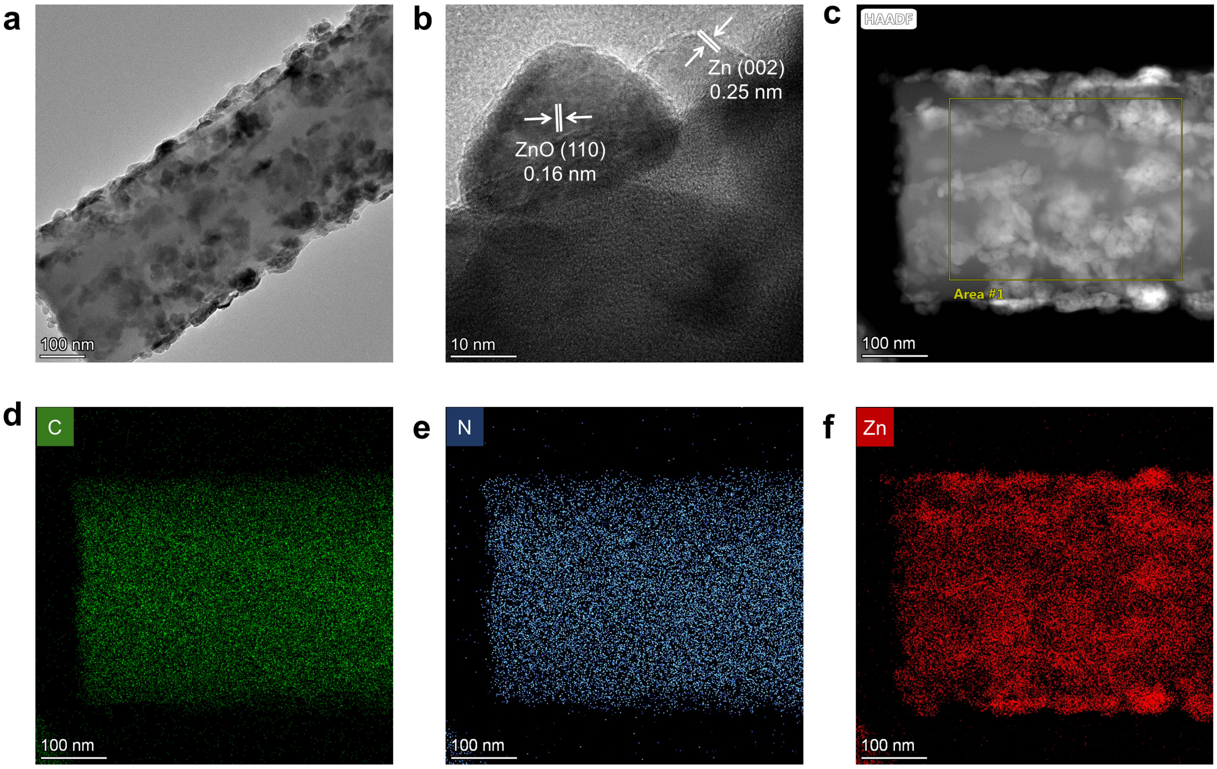

Supplementary Figure 14. Structure of cPAN-Zn. (a) TEM and (b) HRTEM image of cPAN-Zn. (b) HAADF image of cPAN-Zn and corresponding (d) C, (e) N and (f) Zn elemental mapping.

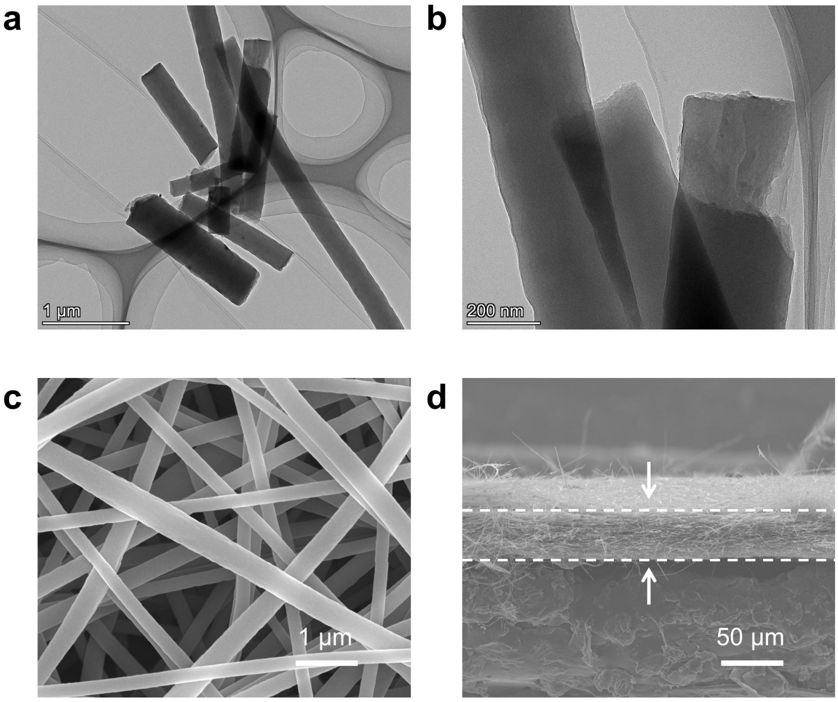

Supplementary Figure 15. Morphology of cPAN. (a, b) TEM images of cPAN. SEM images of (c) front view and (d) cross-section view of cPAN.

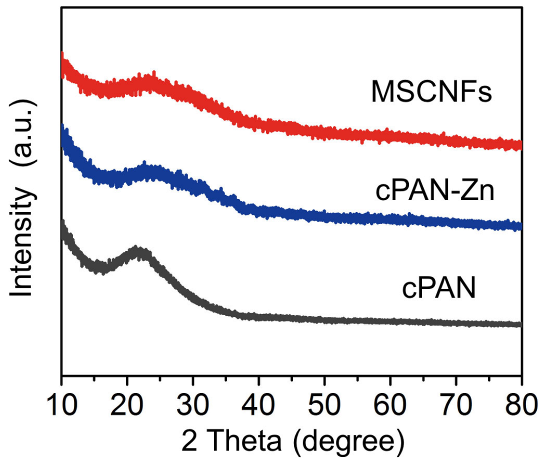

Supplementary Figure 16. Phase characterization of different K metal hosts. PXRD patterns of MSCNFs, cPAN-Zn and cPAN.

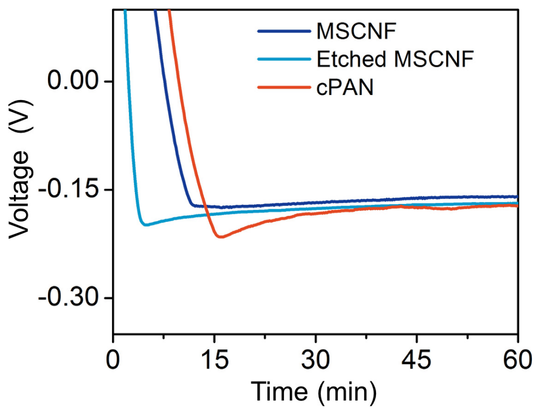

Supplementary Figure 17. Potassiophilicity evaluation. K nucleation behaviour on different K metal hosts at 0.5 mA cm^{-2}.

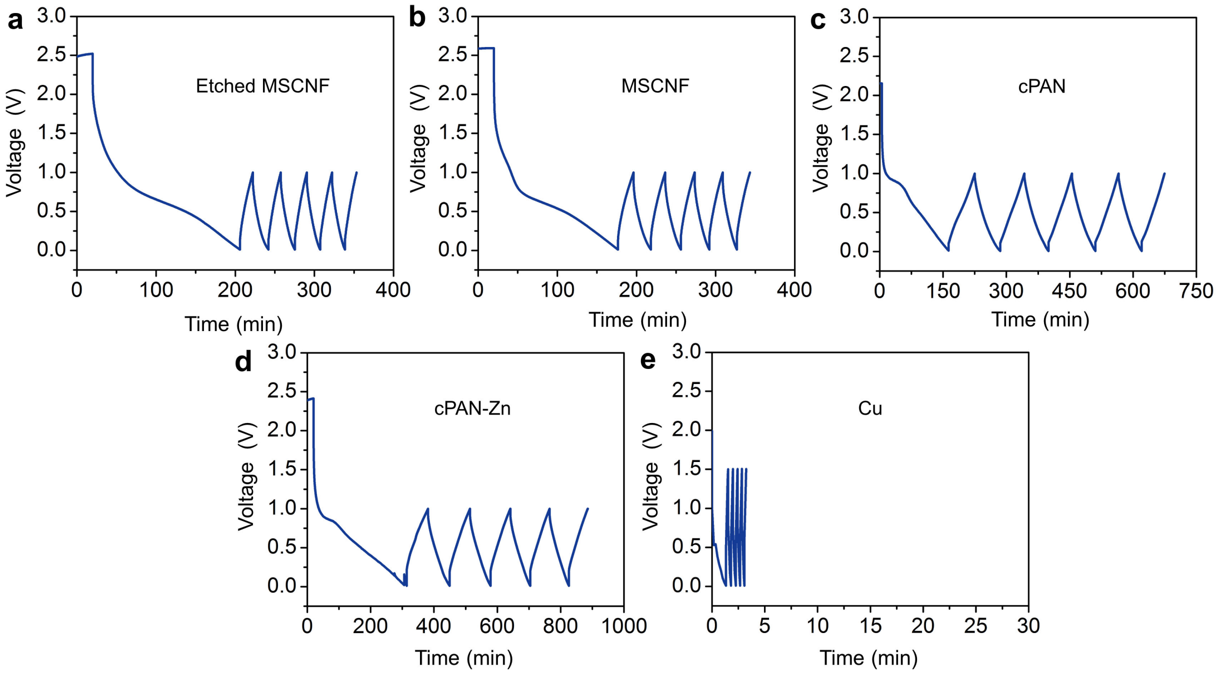

Supplementary Figure 18. Pretreatment of different hosts before plating tests. Formation cycling profiles of (a) etched MSCNF, (b) MSCNF, (c) cPAN, (d) cPAN-Zn and (e) the baseline Cu foil with the voltage range of 0.01–1 V at 0.1 mA cm^{-2}.

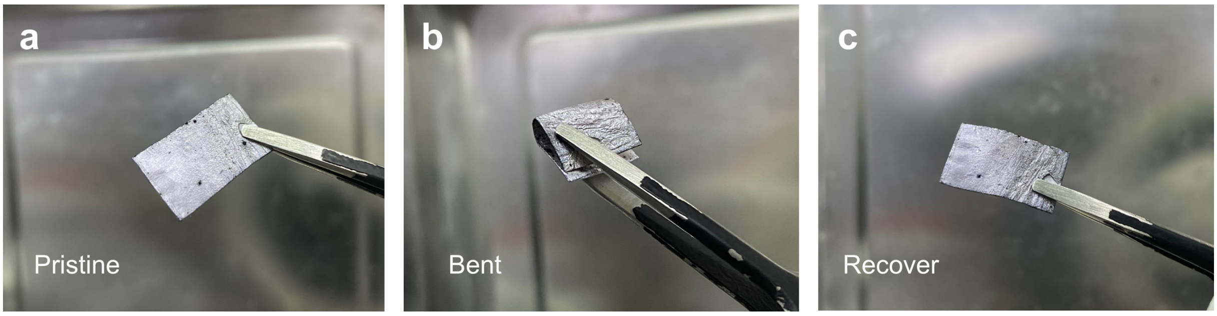

Supplementary Figure 19. Flexibility demonstration of MSCNF-K. Digital photos of (a) pristine, (b) bent and (c) recover state of the infused MSCNF-K anode. The demonstration was conducted in an Ar-filled glovebox with both water and oxygen content lower than 0.1 ppm.

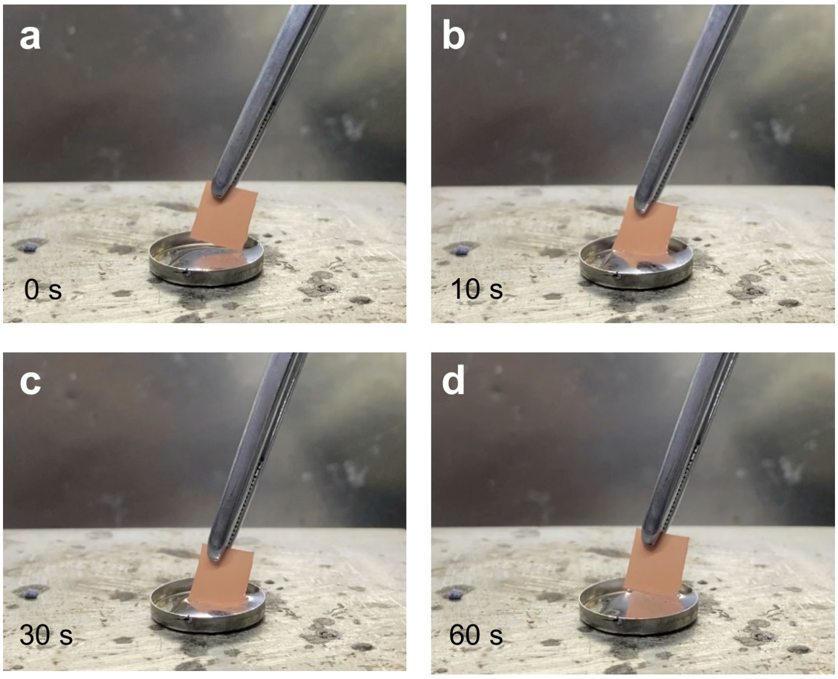

Supplementary Figure 20. K infusion experiment. (a) 0s, (b) 10s, (c) 30s and (d) 60s after the contact of Cu foil to the melted K at 150 °C. The demonstration was conducted in an Ar-filled glovebox with both water and oxygen content lower than 0.1 ppm.

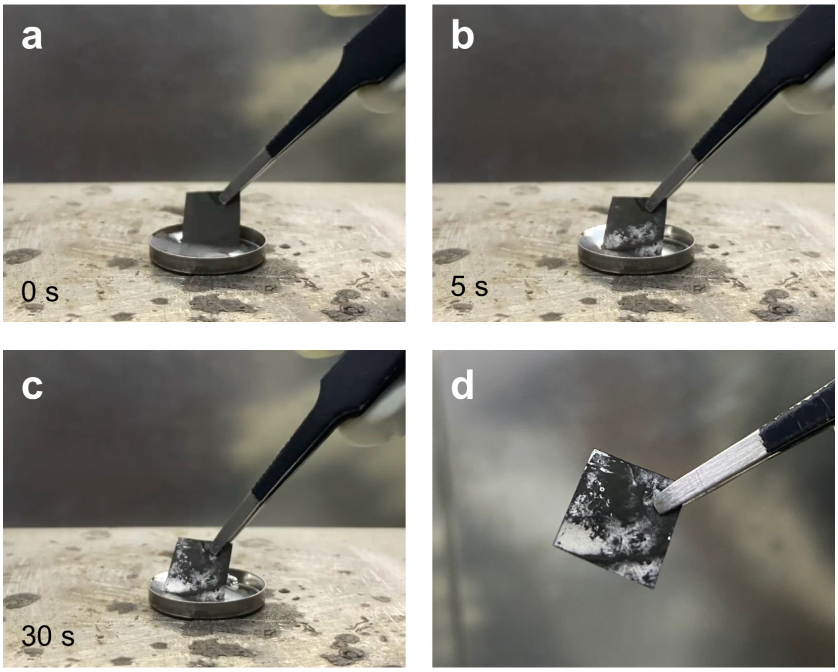

Supplementary Figure 21. K infusion experiment. (a) 0s, (b) 5s and (c) 30s after the contact of cPAN to the melted K at 150 °C. (d) the as-infused cPAN-K composite. The demonstration was conducted in an Ar-filled glovebox with both water and oxygen content lower than 0.1 ppm.

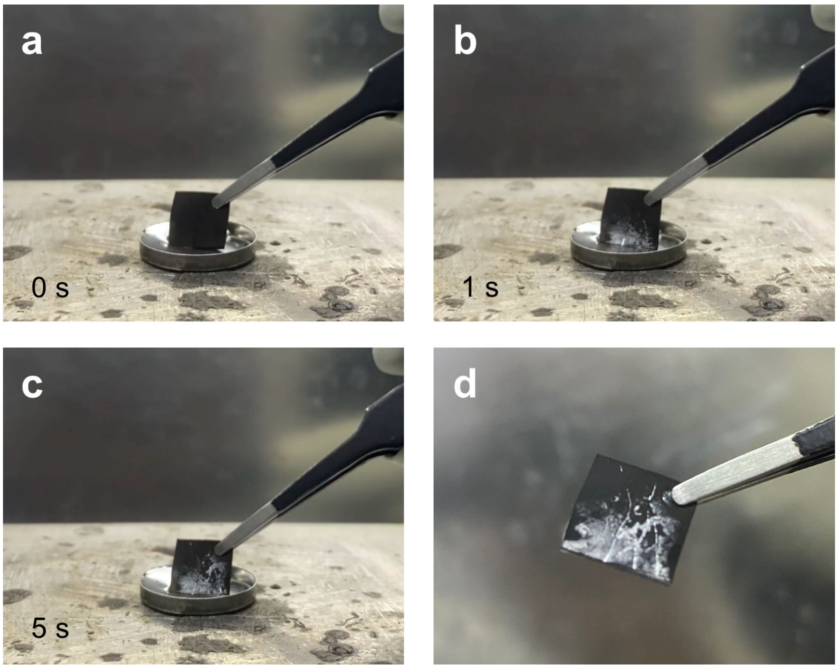

Supplementary Figure 22. K infusion experiment. (a) 0s, (b) 1s and (c) 5s after the contact of cPAN-Zn to the melted K at 150 °C. (d) the as-infused cPAN-Zn-K composite. The demonstration was conducted in an Ar-filled glovebox with both water and oxygen content lower than 0.1 ppm.

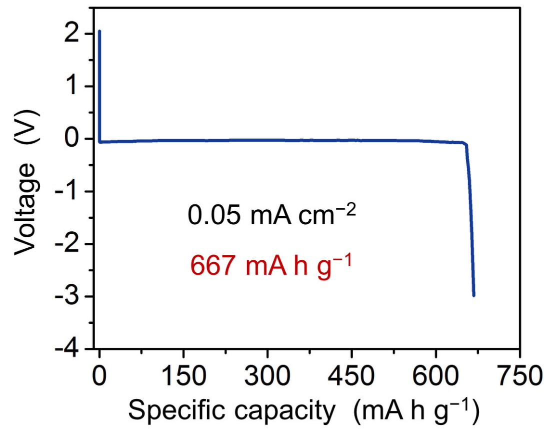

Supplementary Figure 23. K stripping experiment. Voltage profile of stripping K metal in the MSCNF-K||Cu cell at a current density of 0.05 mA cm^{-2}. The capacity was calculated based on the mass of the MSCNF-K composite.

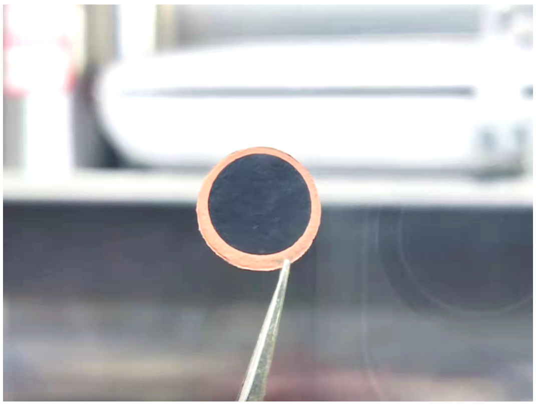

Supplementary Figure 24. K stripping experiment. Digital photo of the MSCNF-K electrode after stripping at a current density of 0.05 mA cm^{-2}. The demonstration was conducted in an Ar-filled glovebox with both water and oxygen content lower than 0.1 ppm.

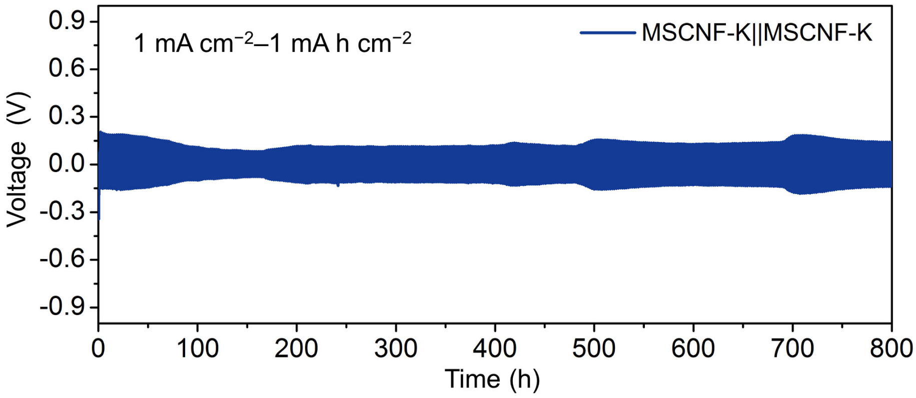

Supplementary Figure 25. Electrochemical performance of symmetric cells. Cycling stability of MSCNF-K||MSCNF-K symmetric cell at a current density of 1 mA cm^{-2} and cut-off capacity of 1 mA h cm^{-2}.

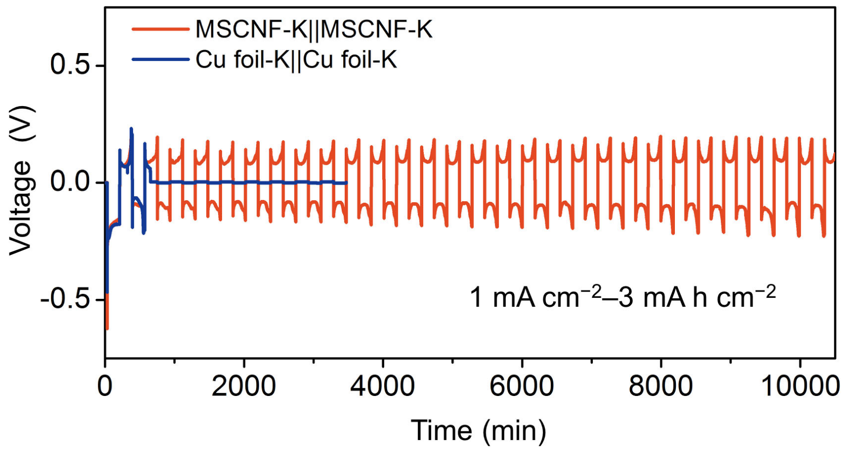

Supplementary Figure 26. Electrochemical performance of symmetric cells. Cycling stability of MSCNF-K||MSCNF-K symmetric cell at a current density of 1 mA cm^{-2} and cut-off capacity of 3 mA h cm^{-2}.

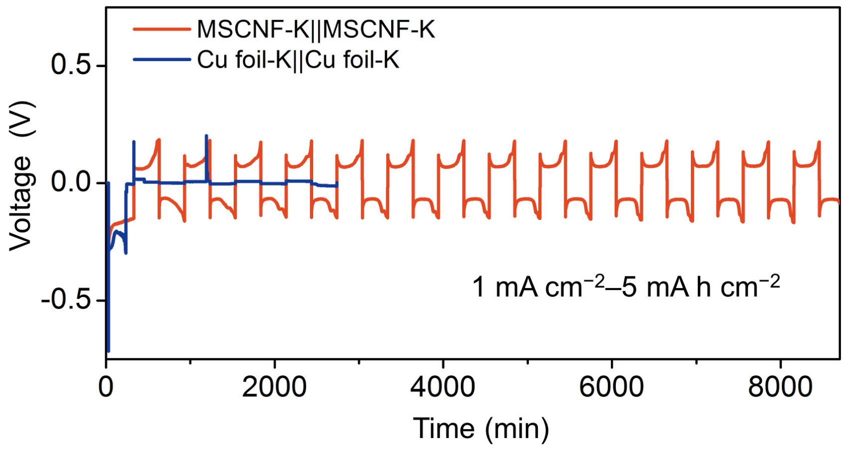

Supplementary Figure 27. Electrochemical performance of symmetric cells. Cycling stability of MSCNF-K||MSCNF-K symmetric cell at a current density of 1 mA cm^{-2} and cut-off capacity of 5 mA h cm^{-2}.

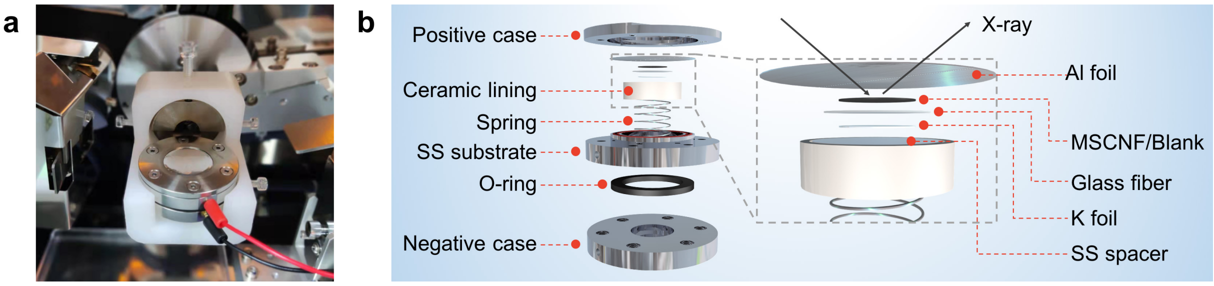

Supplementary Figure 28. Demonstration of the operando XRD cell. (a) Digital image and (b) schematic of the operando XRD cell setup employed in this study.

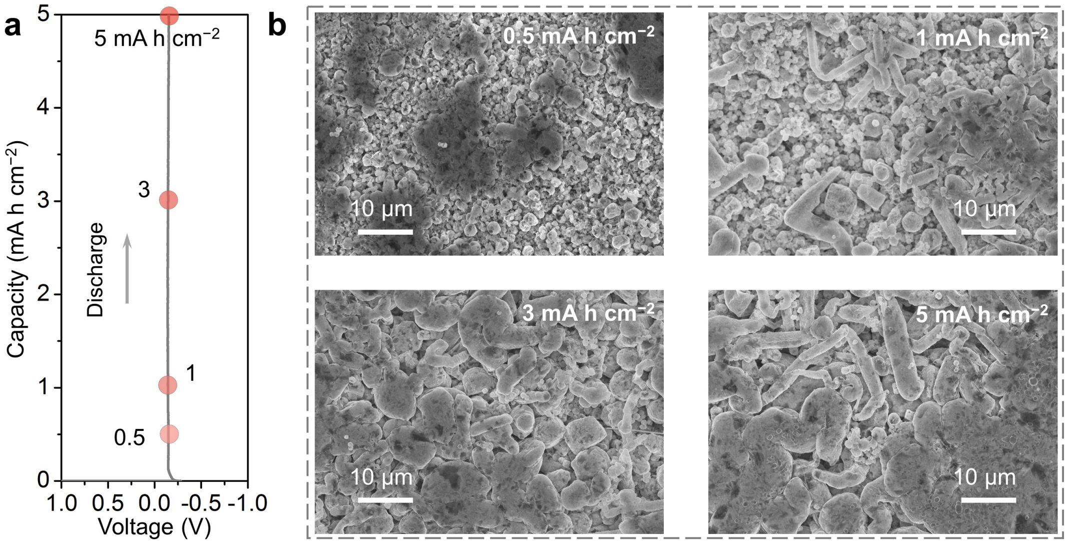

Supplementary Figure 29. Morphology characterization of the plated Cu foil. (a) Voltage profile of K deposition on Cu foil at a current density of 0.5 mA cm^{-2}. (b) SEM images of the corresponding surface morphology at different discharge states. All electrochemical measurements were carried out at a temperature of 25 ± 2 °C.

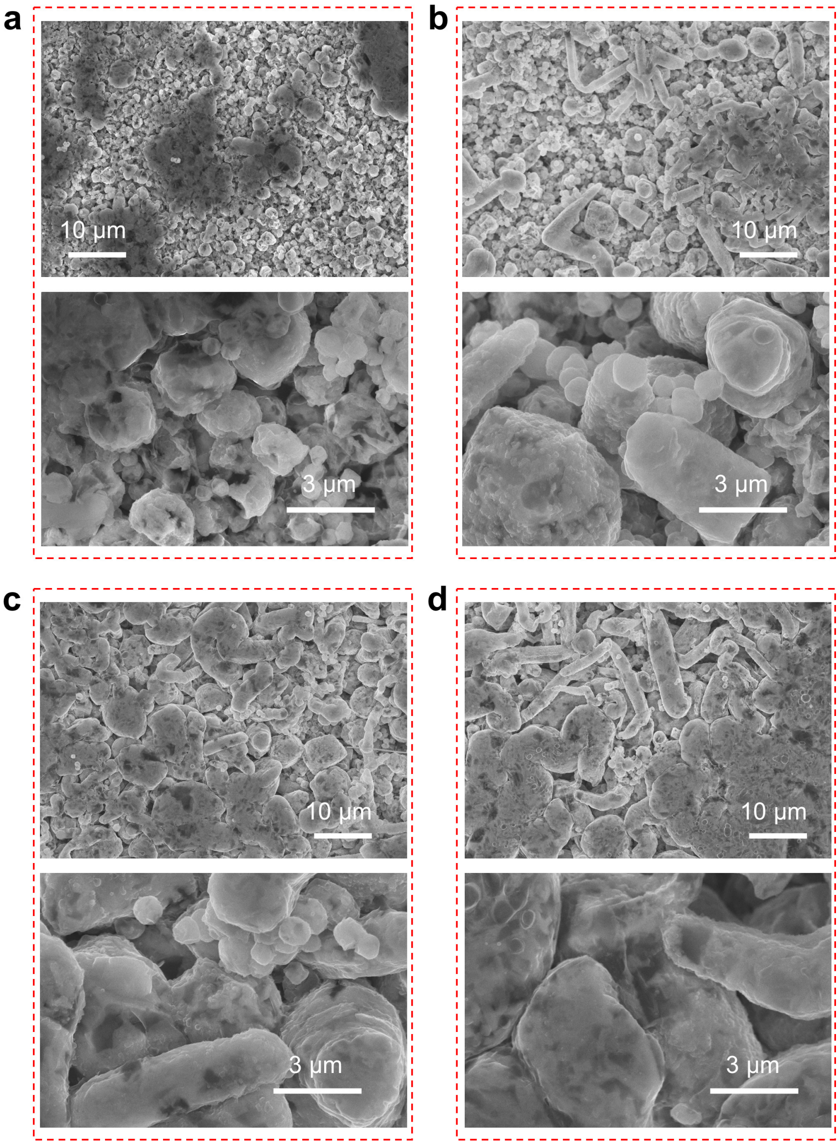

Supplementary Figure 30. Morphology characterization of the plated Cu foil. SEM images of the surface morphology of K deposited on Cu foil at the capacity of (a) 0.5 mA h cm^{-2}, (b) 1 mA h cm^{-2}, (c) 3 mA h cm^{-2} and (d) 5 mA h cm^{-2} (top) and corresponding enlarged images (bottom). The electrodes were harvested from the Cu||K half cells at discharged states. All electrochemical measurements were carried out at a temperature of 25 ± 2 °C.

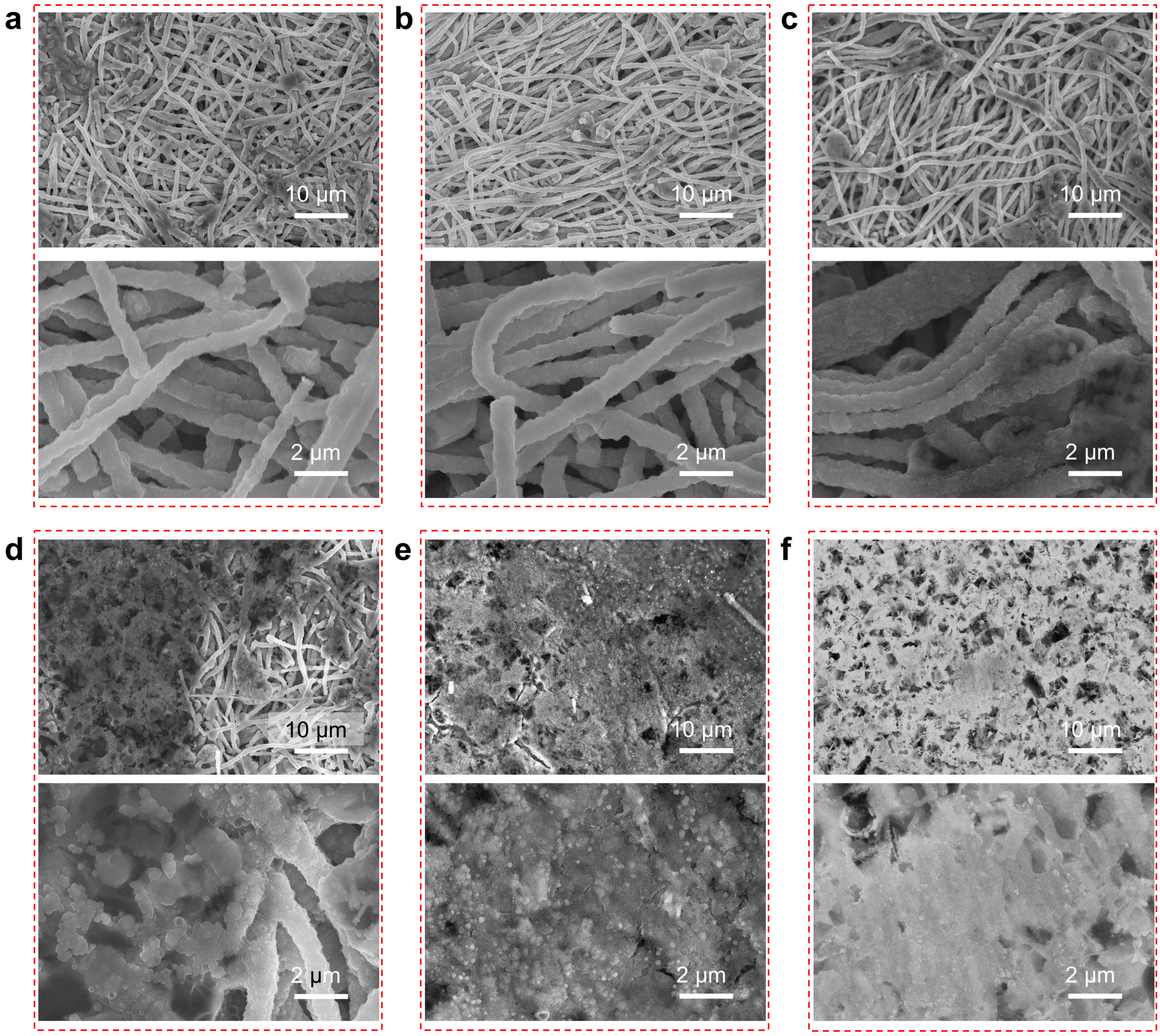

Supplementary Figure 31. Morphology characterization of the plated MSCNF. SEM images of the surface morphology of K deposited on MSCNFs at the capacity of (a) 0.5 mA h cm^{-2}, (b) 1 mA h cm^{-2}, (c) 3 mA h cm^{-2}, (d) 5 mA h cm^{-2}, (e) 8 mA h cm^{-2} and (d) 10 mA h cm^{-2} (top) and corresponding enlarged images (bottom). The electrodes were harvested from the MSCNF||K half cells at discharged states. All electrochemical measurements were carried out at a temperature of 25 ± 2 °C.

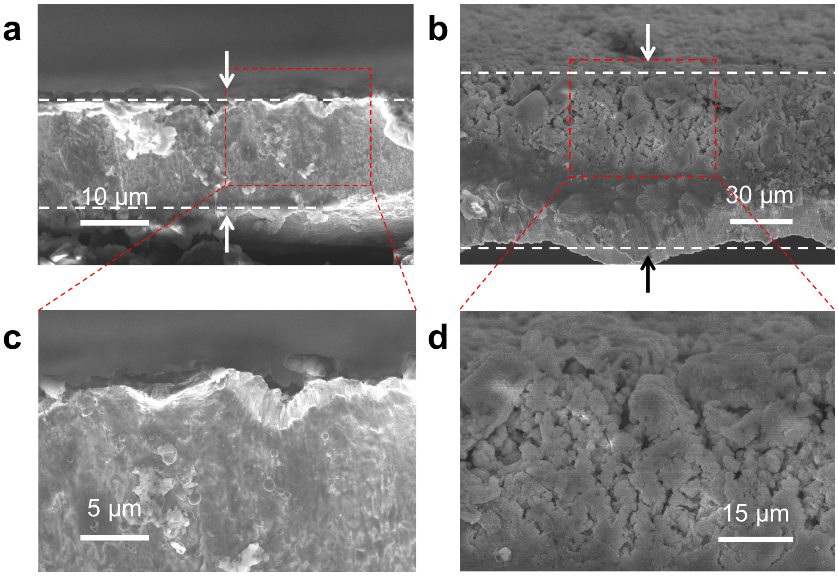

Supplementary Figure 32. Morphology characterization of the plated Cu foil. SEM images of the cross-section view of K deposited on Cu foil at the capacity of (a) 0.5 mA h cm^{-2} and (b) 3 mA h cm^{-2}. (c, d) Corresponding enlarged images. The electrodes were harvested from the Cu||K half cells at discharged states. All electrochemical measurements were carried out at a temperature of 25 ± 2 °C.

Theoretically, the relationship between deposition capacity and the thickness of K plated on a flat substrate can be described *via* the following equation:

$$\mathrm{T} = \frac{QM}{zF} \times \frac{1}{\rho} \tag{1}$$

where T is the thickness, Q is the areal capacity (mA h cm^{-2}), M is the molar mass (g mol^{-1}), z is the number of electrons transferred during K plating (in this case, z = 1), F is the Faraday constant (C mol^{-1}), ρ is the density (g cm^{-3}). Accordingly, it can be calculated that a K deposition capacity of 0.5, 3 and 5 mA h cm^{-2} corresponds to a thickness of 8.5, 50.7 and 84.4 μm, respectively. For example, when the deposition capacity is 3 mA h cm^{-2}, the corresponding thickness can be calculated as follows:

$$\mathrm{T} = \frac{3\ \mathrm{mA\ h\ cm^{-2}} \times 39.1\ \mathrm{g\ mol^{-1}}}{96485\ \mathrm{C\ mol^{-1}}} \times \frac{1}{0.862\ \mathrm{g\ cm^{-3}}}$$

$$= \frac{3\times 3.6\ \mathrm{C\ cm^{-2}} \times 39.1\ \mathrm{g\ mol^{-1}}}{96485\ \mathrm{C\ mol^{-1}}} \times \frac{1}{0.862\ \mathrm{g\ cm^{-3}}} \approx 5.07\times 10^{-3}\ \mathrm{cm} = 50.7\ \mu\mathrm{m}$$

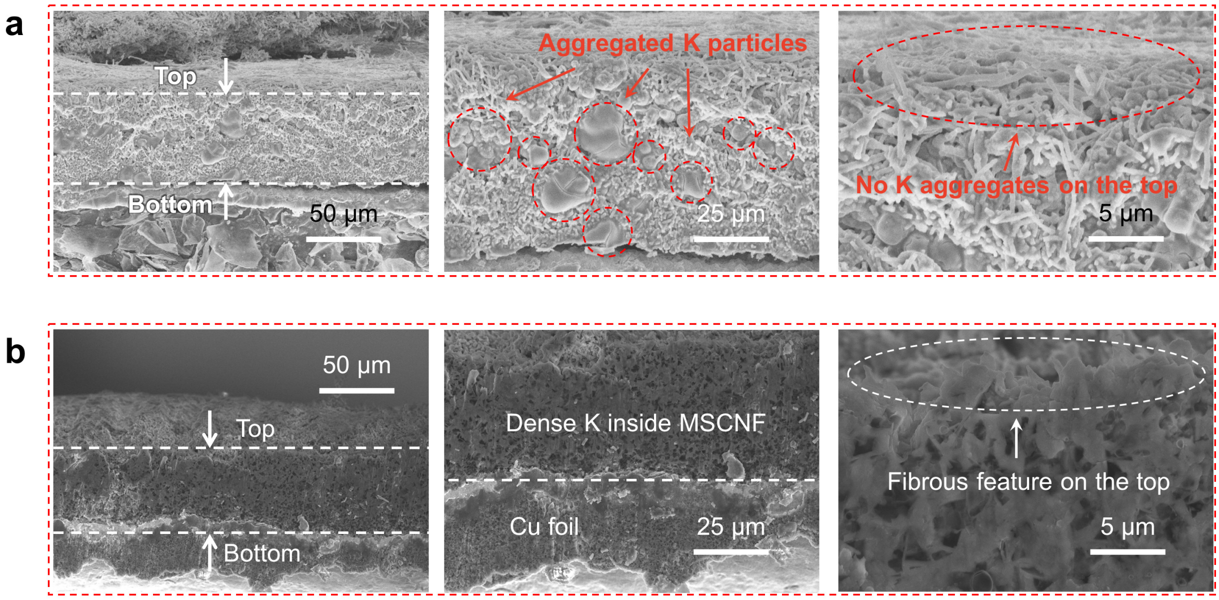

Supplementary Figure 33. Morphology characterization of the plated MSCNF. SEM images of the cross-section view of K deposited on MSCNFs at the capacity of (a) 0.5 mA h cm^{-2} and (b) 3 mA h cm^{-2}. The electrodes were harvested from the MSCNF||K half cells at discharged states. All electrochemical measurements were carried out at a temperature of 25 ± 2 °C.

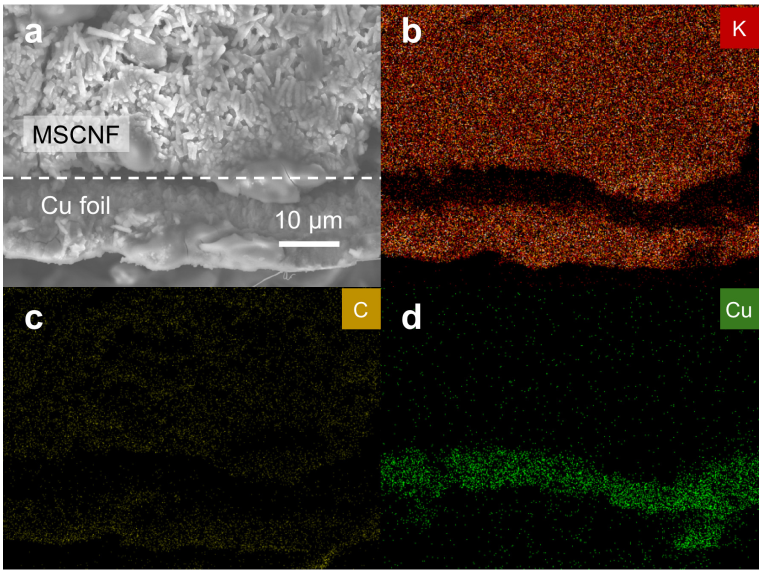

Supplementary Figure 34. Morphology characterization of the plated MSCNF. (a) SEM image of the cross-section view of K deposited on MSCNFs at the capacity of 0.5 mA h cm^{-2} and corresponding elemental mapping of (b) K, (c) C and (d) Cu. The electrodes were harvested from the Cu||K half cells at discharged states. All electrochemical measurements were carried out at a temperature of 25 ± 2 °C.

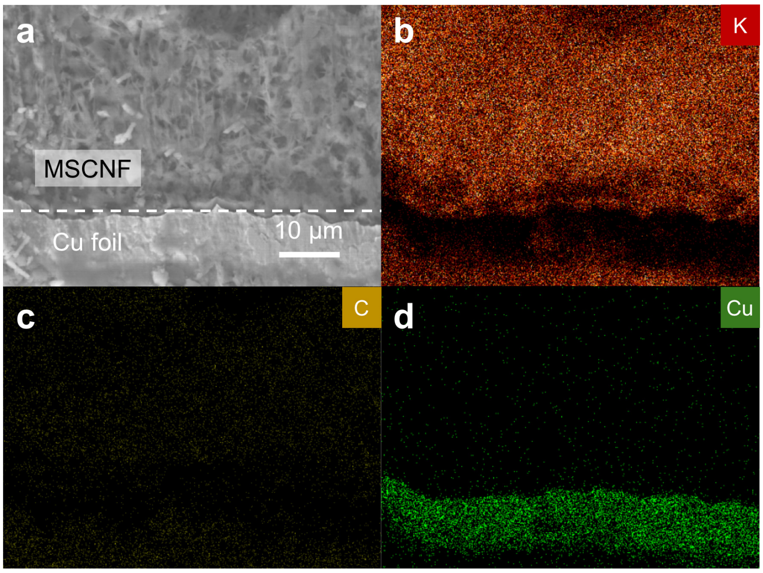

Supplementary Figure 35. Morphology characterization of the plated MSCNF. (a) SEM image of the cross-section view of K deposited on MSCNFs at the capacity of 3 mA h cm^{-2} and corresponding elemental mapping of (b) K, (c) C and (d) Cu. The electrodes were harvested from the MSCNF||K half cells at discharged states. All electrochemical measurements were carried out at a temperature of 25 ± 2 °C.

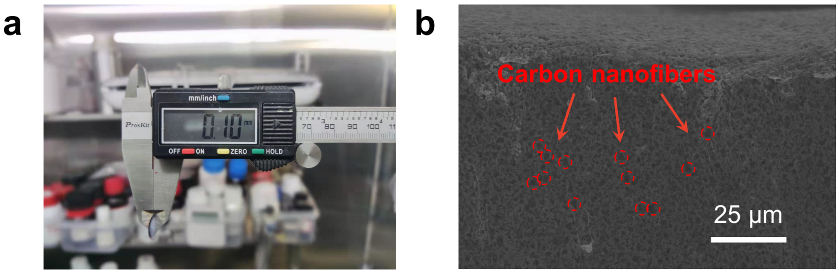

Supplementary Figure 36. Morphology characterization of the plated MSCNF. (a) Digital photo of thickness measurement (the demonstration was conducted in an Ar-filled glovebox with both water and oxygen content lower than 0.1 ppm) and (b) cross-sectional SEM image of a MSCNF host deposited with a capacity of 5 mA h cm^{-2}. The electrodes were harvested from the MSCNF||K half cells at discharged states. All electrochemical measurements were carried out at a temperature of 25 ± 2 °C.

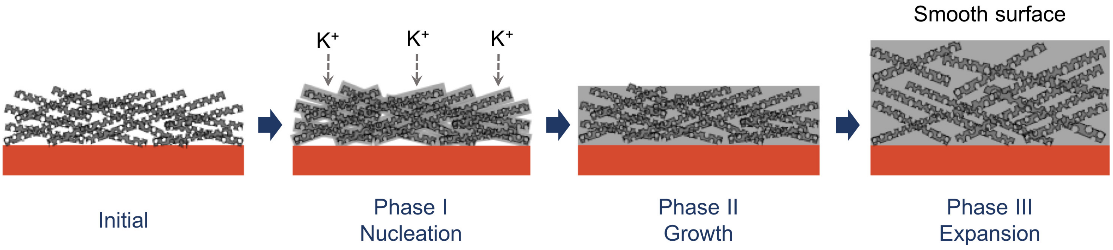

Supplementary Figure 37. Schematic illustration of K plating on MSCNF.

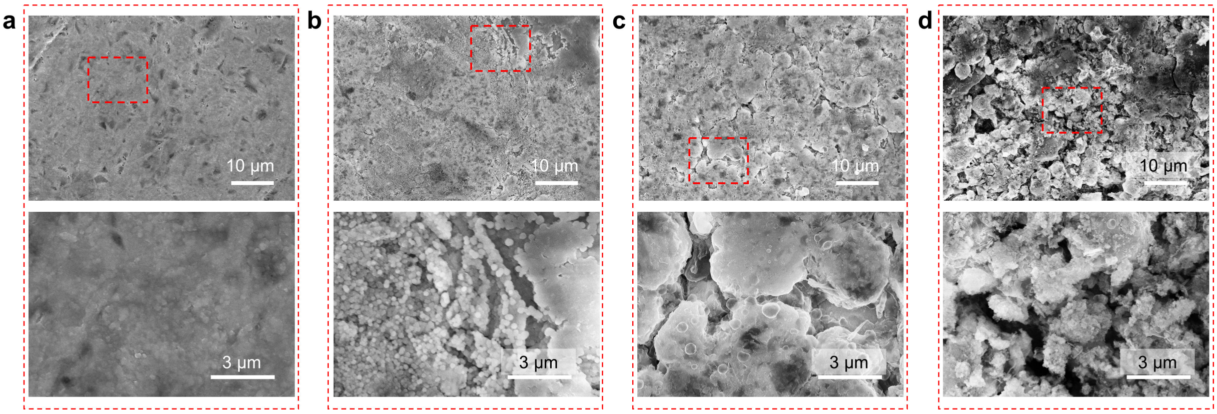

Supplementary Figure 38. Morphology characterization of bare K anode. SEM images (top) and corresponding enlarged images (bottom) of the surface morphology of Cu-K electrodes harvested from cycled Cu-K||Cu-K symmetric cells (1 mA cm^{-2} and 1 mA h cm^{-2}) after (a) 0, (b) 1, (c) 10 and (d) 50 cycles. All electrochemical measurements were carried out at a temperature of 25 ± 2 °C.

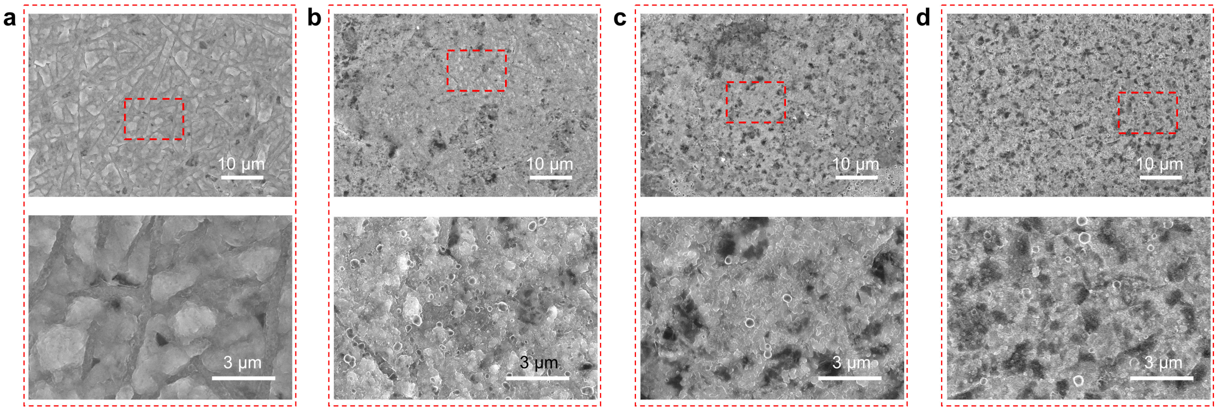

Supplementary Figure 39. Morphology characterization of MSCNF-K anode. SEM images and corresponding enlarged images of the surface morphology of MSCNF-K electrodes harvested from cycled MSCNF-K||MSCNF-K symmetric cells (1 mA cm^{-2} and 1 mA h cm^{-2}) after (a) 0, (b) 1, (c) 10 and (d) 50 cycles. All electrochemical measurements were carried out at a temperature of 25 ± 2 °C.

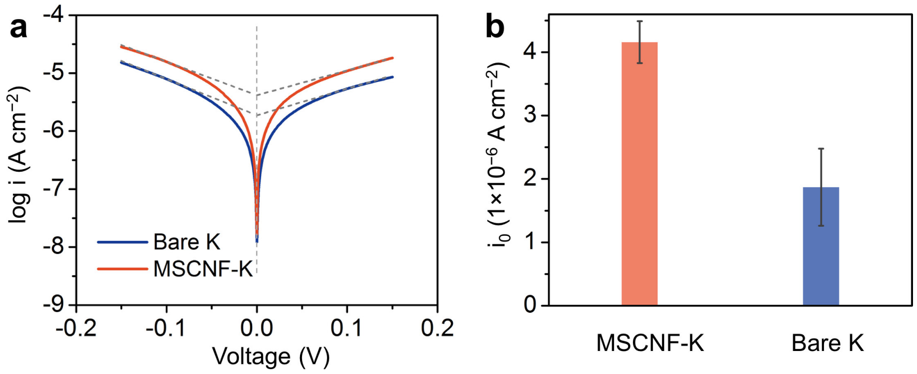

Supplementary Figure 40. Electrochemical characterization of different K metal anodes. (a) Tafel plots obtained from LSV results. (b) Comparison of exchange current densities (I_0). The error bars represent the standard deviation.

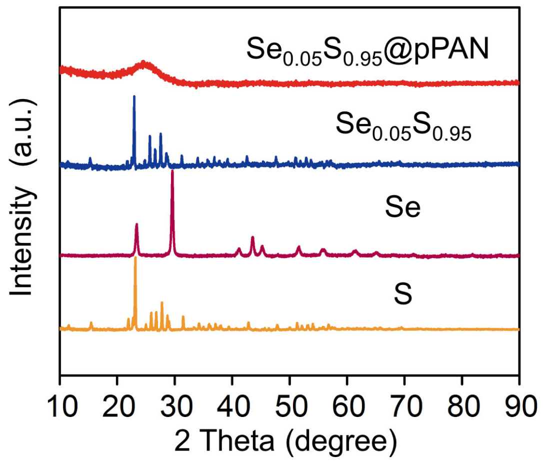

Supplementary Figure 41. Phase characterization of different powder samples. XRD patterns of $Se_{0.05}S_{0.95}$@pPAN, $Se_{0.05}S_{0.95}$, Se and S.

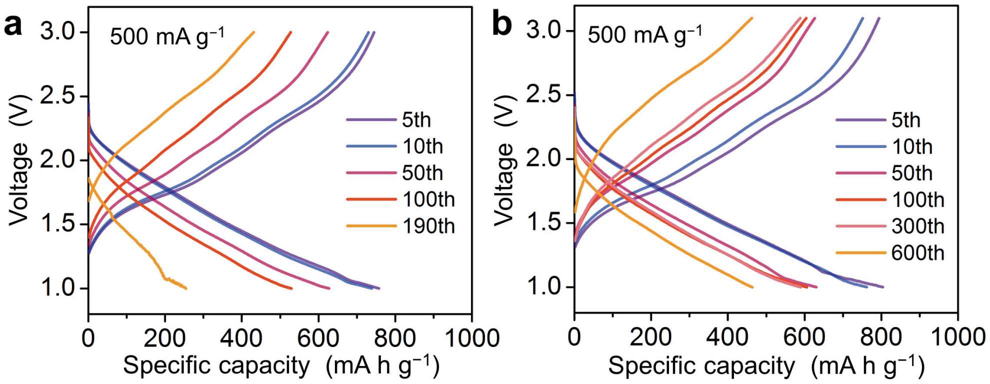

Supplementary Figure 42. Electrochemical performance of K–S full cells. Voltage profiles of (a) Cu-K||$Se_{0.05}S_{0.95}$@pPAN cell and (b) MSCNF-K||$Se_{0.05}S_{0.95}$@pPAN cell at different cycles under the specific current of 500 mA g^{-1}. All electrochemical measurements were carried out at a temperature of 25 ± 2 °C.

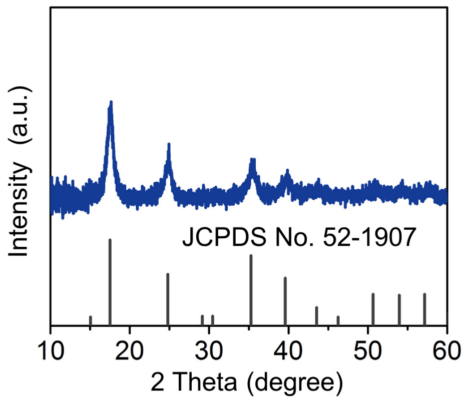

Supplementary Figure 43. Phase characterization of PPB. XRD patterns of as-synthesized PPB powder.

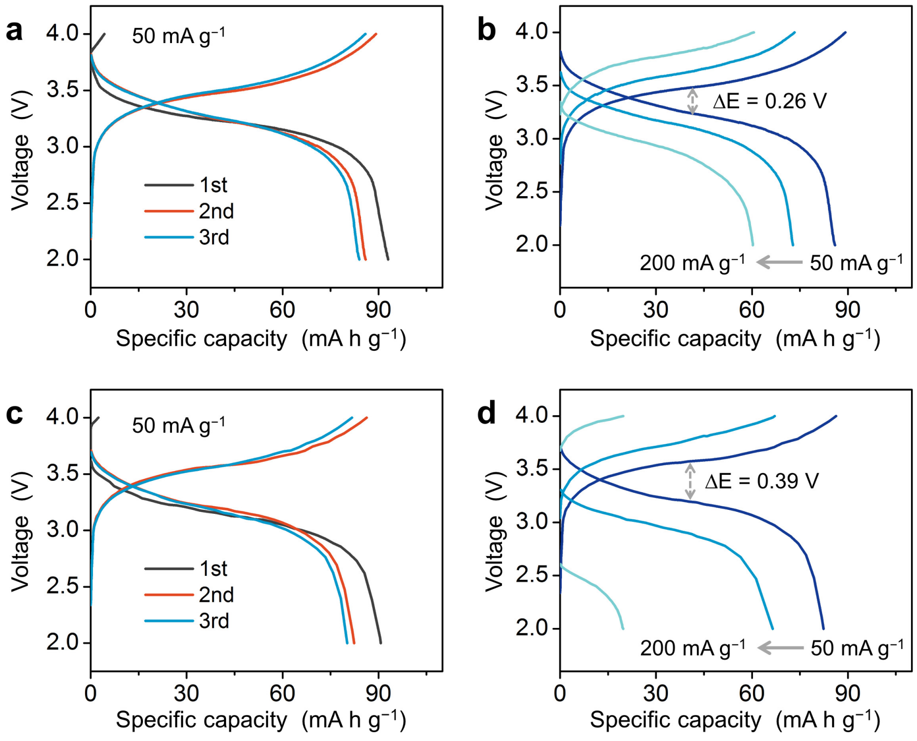

Supplementary Figure 44. Electrochemical performance of K-PPB full cells. Voltage profiles of (a,b) MSCNF-K||PPB and (c,d) Cu-K||PPB full-cells at (a,c) different cycles and (b,d) different specific current rates (50, 100 and 200 mA g^{-1}). All electrochemical measurements were carried out at a temperature of 25 ± 2 °C.

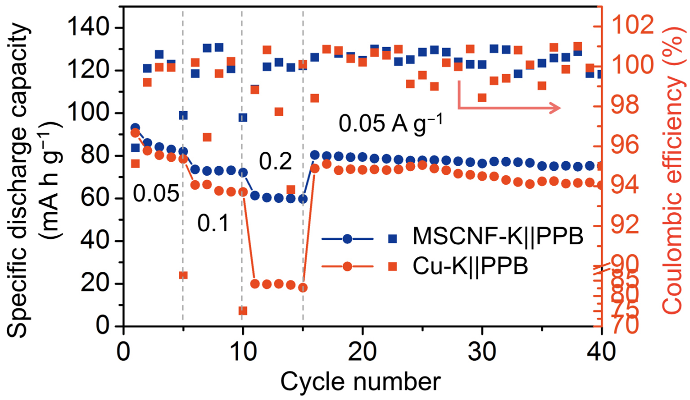

Supplementary Figure 45. Electrochemical performance of K-PPB full cells. Rate performance of MSCNF-K||PPB and Cu-K||PPB full-cells. All electrochemical measurements were carried out at a temperature of 25 ± 2 °C.

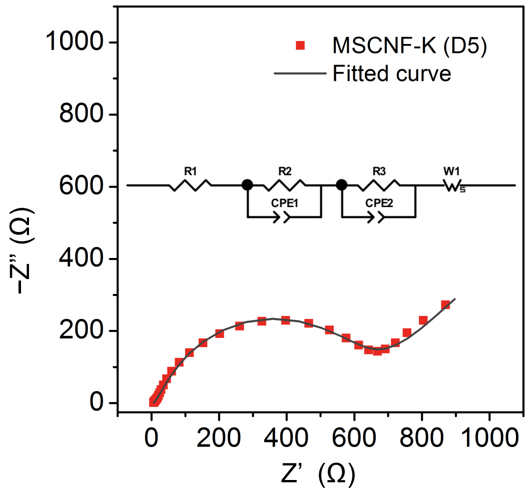

Supplementary Figure 46. Electrochemical impedance spectroscopy characterization. Experimental and simulation EIS curve of the MSCNF-K||$Se_{0.05}S_{0.95}$@pPAN cell at certain state of charge. Inset is the corresponding equivalent circuit diagram.

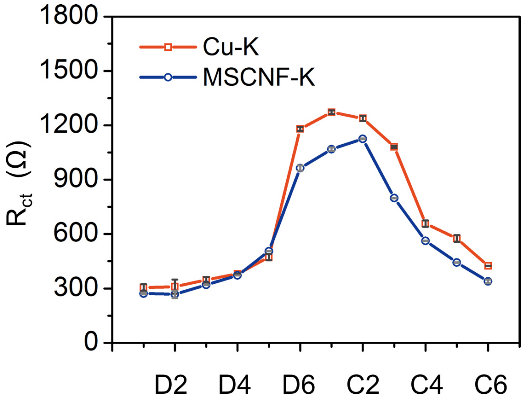

Supplementary Figure 47. Electrochemical impedance spectroscopy characterization. R_{ct} value obtained from the in situ EIS of different K composite anode at different state of charge (D2: discharged at 2.4 V; D4: discharged at 1.6 V; D6: discharged at 1.0 V; C2: recharged at 1.6 V; C4: recharged at 2.3 V; C6: recharged at 3.0 V). The error bars represent the standard deviation.

Supplementary Table 1. Surface areas and pore volumes of different hosts.

	S_{BET} (m^2 g^{-1})	V_{total} (cm^3 g^{-1})	Error (%)
cPAN	27	0.04	3.91
cPAN-Zn	30	0.05	3.44
MSCNFs	82	0.23	0.66

Supplementary Table 2. Elemental composition of different hosts based on XPS analysis.

	C (wt%)	Error (%)	N (wt%)	Error (%)	O (wt%)	Error (%)	Zn (wt%)	Error (%)
cPAN	76.3	1.1	15.3	7.9	8.4	21.2	/	/
cPAN-Zn	52.9	1.9	14.3	10.6	7.9	15.1	24.9	3.9
MSCNFs	60.1	2.4	15.2	10.5	6.9	18.2	17.8	3.7
Etched MSCNFs	76.8	1.5	18.4	11.6	4.8	22.6	/	/

Supplementary Table 3. Performance of existing K metal hosts.

	Areal mass (mg cm^{-2})	Gravimetric capacity (mA h g^{-1}) [(a)]	Nucleation overpotential (mV)	Thickness (μm)	Infusion time (s cm^{-2})	Infusion temperature (°C)	Ref.
**MSCNFs**	**0.5**	**667 (97%)**	**15** (0.5 mA cm^{-2})[(b)]	**100**	**1**	**150**	**This work**
ACM	1.6	516 (75%)	94 (1 mA cm^{-2})	150	19.48	~100	1
HNCP/G	N/A	N/A	N/A	100	N/A	N/A	2
rGO@3D-Cu	N/A	N/A	139 (0.5 mA cm^{-2})	200	5.31	300	3
Cu_3Pt–Cu mesh	N/A	586 (85%)	30 (0.1 mA cm^{-2})	N/A	N/A	N/A	4
DN-MXene/CNT	4	428 (70%)	84 (0.5 mA cm^{-2})	150	16.67	>70	5
PM/NiO	N/A	N/A	30 (0.4 mA cm^{-2})	N/A	N/A	N/A	6
PCNF@SnO_2	N/A	598 (87%)	13 (0.5 mA cm^{-2})	N/A	265.49	~250	7
Al@Al-D	N/A	N/A	490 (0.5 mA cm^{-2})	N/A	N/A	N/A	8

[(a)] Gravimetric capacity is calculated based on the total mass of the host and K metal in the anode. The % values in brackets mean the proportion of the reported gravimetric discharge capacity value to the theoretical value of pure K metal (687 mA h g^{-1}).

[(b)] The current density values in brackets refer to the applied current densities at which the corresponding nucleation overpotentials were obtained.

Supplementary Table 4. The elemental analysis of $Se_{0.05}S_{0.95}$@pPAN.

	C (wt%)	N (wt%)	S (wt%)	Se (wt%)	Se_xS_{1-x} molar ratio	Se&S in the composite (wt%)
Value	36.91	13.39	43.51	4.98	0.05	48.49
Error (%)	0.94	4.48	0.66	9.12	/	/

Supplementary Table 5. Fitted impedance parameters of the K-S cells based on Cu-K and MSCNF-K anodes under different states of charge.

State of charge	Equivalent circuit	Cu-K						MSCNF-K					
		R_s (Ω)	Error (%)	R_f (Ω)	Error (%)	R_{ct} (Ω)	Error (%)	R_s (Ω)	Error (%)	R_f (Ω)	Error (%)	R_{ct} (Ω)	Error (%)
D1 (OCV)		6.8	7.3	51.6	6.0	304.7	6.3	6.3	7.9	41.0	0.2	272.0	1.7
D2 (2.3 V)		6.8	8.8	54.7	1.8	309.9	10.5	6.4	10.9	46.9	0.4	267.8	7.7
D3 (2.0 V)		6.7	7.4	61.3	1.6	347.6	4.3	6.4	10.0	58.0	0.2	319.4	0.2
D4 (1.6 V)		6.8	10.7	73.7	1.4	380.1	0.1	6.4	9.4	60.7	0.5	371.7	0.1
D5 (1.3 V)		6.8	10.7	83.7	1.7	472.8	3.6	6.4	10.1	64.0	1.6	505.7	0.1
D6 (1.0 V)	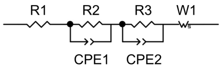	6.9	4.3	96.5	10.7	1180	0.9	6.6	6.1	83.6	10.0	964	10.8
C1 (1.3 V)		7.2	6.9	105.4	10.1	1272	0.8	6.8	5.9	84.3	0.4	1068	1.0
C2 (1.6 V)		7.2	9.7	109.1	9.2	1238	0.1	6.7	1.0	70.7	0.7	1125	0.2
C3 (2.0 V)		7.0	10.2	101.0	4.1	1081	0.5	6.6	10.9	60.2	0.3	798.6	0.1
C4 (2.3 V)		6.9	10.4	99.6	0.3	657.5	2.7	6.6	10.2	56.4	0.4	562.6	0.2
C5 (2.6 V)		6.9	5.8	83.0	8.6	575.2	3.2	6.5	7.7	49.7	10.7	443.0	0.2
C6 (3.0 V)		6.8	7.4	64.2	2.3	424.3	0.2	6.4	10.6	49.0	10.1	338.8	3.0

References

1 Qin L., *et al.* Capillary Encapsulation of Metallic Potassium in Aligned Carbon Nanotubes for Use as Stable Potassium Metal Anodes. *Adv. Energy Mater.* **9**, 1901427 (2019).

2 Ye M., Hwang J. Y., Sun Y. K. A 4 V Class Potassium Metal Battery with Extremely Low Overpotential. *ACS Nano* **13**, 9306-9314 (2019).

3 Liu P., *et al.* Stable Potassium Metal Anodes with an All-Aluminum Current Collector through Improved Electrolyte Wetting. *Adv. Mater.* **32**, 2002908 (2020).

4 Wang J., *et al.* Cu3Pt alloy-functionalized Cu mesh as current collector for dendritic-free anodes of potassium metal batteries. *Nano Energy* **75**, 104914 (2020).

5 Tang X., *et al.* MXene-Based Dendrite-Free Potassium Metal Batteries. *Adv. Mater.* **32**, 1906739 (2020).

6 Li Y., *et al.* Original growth mechanism for ultra-stable dendrite-free potassium metal electrode. *Nano Energy* **62**, 367-375 (2019).

7 Zhao X., *et al.* Enhanced surface binding energy regulates uniform potassium deposition for stable potassium metal anodes. *J. Mater. Chem. A* **8**, 5671-5678 (2020).

8 Liu P., *et al.* Stable Potassium Metal Anodes with an All-Aluminum Current Collector through Improved Electrolyte Wetting. *Adv. Mater.* **32**, 2002908 (2020).
