# Supplementary material for: Codoped porous carbon nanofibres as a potassium metal host for nonaqueous K-ion batteries
Source: Nat Commun. 2022 Aug 20;13:4911. doi: 10.1038/s41467-022-32660-y (PMC9392754; doi:10.1038/s41467-022-32660-y)
Supplement: Supplementary file 3 — Description of additional Supplementary File [file 41467_2022_32660_MOESM3_ESM.pdf]

Descriptions of Additional Supplementary Files

Supplementary Movie 1. K infusion experiment on MSCNFs.

Supplementary Movie 2. K infusion experiment on Cu foils.

Supplementary Movie 3. K infusion experiment on cPAN.

Supplementary Movie 4. K infusion experiment on cPAN-Zn.
